# pyMSscreen: an interactive interface for automated quality control and prescreening in mass spectrometry workflows

**DOI:** 10.1186/s13321-026-01260-z

**Published:** 2026-08-10

**Authors:** Anjana Elapavalore, Parviel Chirsir, Emma H. Palm, Martin Jakubec, Emma L. Schymanski

**Affiliations:** Luxembourg Centre for Systems Biomedicine (LCSB), University of Luxembourg, 6 Avenue du Swing, 4367 Belvaux, Luxembourg

**Keywords:** High resolution mass spectrometry, Quality control, Interactive visualisation, Spectral data analysis, Non-target screening, Open source software, Docker

## Abstract

Reliable quality control of mass spectrometry data remains a major bottleneck in target, suspect and non-target screening workflows. In non-target analysis, thousands of features can be extracted through automated peak picking, many of which require manual verification to exclude artefacts arising from noise, co-elution, inconsistent chromatographic behavior or statistical prioritization. Similarly, suspect screening workflows depend on effective chromatographic and spectral quality control to support confidence in annotated features. However, existing quality control practices are often fragmented across vendor software and custom scripts, limiting reproducibility and scalability. This article describes pyMSscreen, a unified framework that enables automated and reproducible chromatographic quality control across target, suspect, and non-target mass spectrometry workflows. The backend leverages RDKit and pyOpenMS for compound validation and spectral extraction, while the frontend provides a web-based interface for systematic inspection of chromatograms and mass spectra. The development of pyMSscreen draws on experience gained from the earlier R-based Shinyscreen, which showed that accessible graphical user interface can greatly simplify the review of liquid chromatography mass spectrometry data. However, the practical limitations of the Shiny framework, including difficulties with deployment, motivated the creation of a more flexible and modern solution. pyMSscreen employs a modular architecture that supports target, suspect, and non-target screening by accommodating both SMILES or *m/z* only input lists. Automated quality control metrics are applied consistently across workflows, enabling assessment of peak shape, signal intensity, retention-time alignment, and background noise prior to downstream interpretation. Container-based deployment ensures reproducible installation and execution across computational environments. The performance and applicability of pyMSscreen is illustrated using a representative non-target dataset and suspect screening workflow demonstrating consistent results relative to established approaches, while substantially reducing manual data handling and review time. Overall, pyMSscreen provides an efficient, transparent, and reproducible platform for chromatographic quality control and data curation in high resolution mass spectrometry based cheminformatics workflows. The code is openly available (Artistic 2.0 license) at https://gitlab.com/uniluxembourg/lcsb/eci/pymsscreen.

**Scientific contribution**

pyMSscreen presents a unified framework for chromatographic quality control that integrates automated quality metrics with interactive visual inspection of mass spectrometry data. The central contribution is the consistent evaluation of chromatographic peak quality and spectral alignment across target, suspect, and non-target screening workflows within a single methodology. This work builds on earlier developments in interactive mass spectrometry data review while establishing a scalable and reproducible approach to feature-level quality control.

**Graphical Abstract:**

**Figure:**
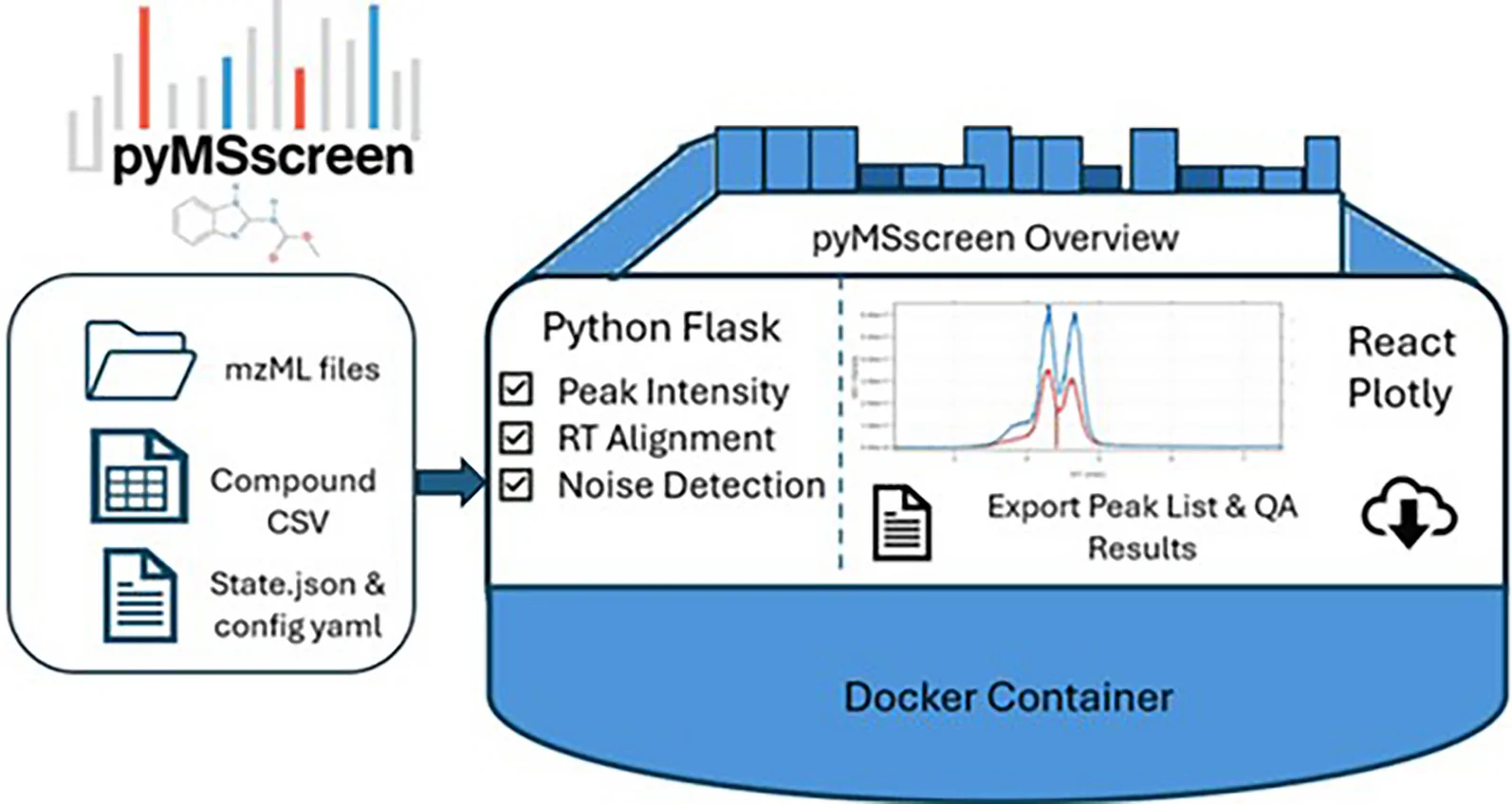

## Introduction

Environmental and metabolomics samples are often highly complex, containing a wide variety of known and unknown chemicals derived from biological processes and/or anthropogenic sources. Many of these substances have the potential to affect human health or disrupt ecological balance, making their detection and characterization a critical task for analytical scientists [1]. Non-target screening (NTS) via liquid chromatography coupled with high-resolution mass spectrometry (LC-HRMS) has become a central analytical approach in the identification of small molecules and chemicals of emerging concern [2]. NTS enables researchers to comprehensively screen complex mixtures and search for both known and unknown chemicals with one acquisition method. Despite its strengths, confidently identifying chemicals in the samples remains challenging, largely due to the sheer number of detected features in each dataset [1]. A typical NTS workflow includes sample collection, preparation, instrumental analysis, and data processing. Of these stages, data processing is often the most challenging bottleneck highlighted by researchers, as it involves several interconnected tasks, including peak detection, alignment, integration, and ultimately linking the observed features to chemical structures [2]. While advanced computational tools have helped to automate many steps, the inherent complexity of data processing continues to impede routine implementation of NTS [1].

To meet these challenges, common data processing workflows in NTS often use a combination of vendor-specific software, open source platforms, and in-house resources. Vendor specific software such as Xcalibur for Thermo instruments [3] provide user-friendly inspection tools but are restricted by licensing, limited compatibility, and a narrow focus on specific instruments. In contrast, open source platforms such as xcms [4], MZmine [5], RforMassSpectrometry [6], and patRoon [7], offer more flexible solutions by allowing users to directly configure and adapt data-processing workflows [8]. However, one key challenge in NTS is not the software itself but the lack of standardized quality control (QC) procedures. Reliable NTS interpretation requires consistent quality metrics, including reproducible signal intensity, adequate signal-to-noise ratios and robust retention-time (RT) alignment between extracted ion chromatograms (EICs) and corresponding tandem mass spectral fragments MS^2^. These QC steps are essential for confirming true chemical signals during screening, yet are often applied inconsistently across workflows. This gap highlights the need for tools that explicitly support high-confidence QC checks, especially for screening-level confirmation based on EIC and MS^2^ peak quality.

One possible solution is the use of Shinyscreen, an open source R Shiny based application, introduced a graphical interface for semi-automated screening, intended to lower the barrier for researchers to perform robust QC without extensive programming experience and enabled rapid validation of MS data [8]. Although the R/Shiny framework of Shinyscreen facilitated rapid prototyping and user interaction, Shiny applications are typically single-threaded, and extending functionality beyond the R environment increases architectural complexity [9]. Although the introduction of a Docker image simplified installation, challenges remained with performance and adaptability.

Building on this foundation and to overcome the limitations of earlier approaches, the current article describes pyMSscreen, a Python-React based framework that extends the functionality of Shinyscreen in a Python-based implementation. pyMSscreen is implemented as a full-stack framework consisting of a Python-based backend and a web-based frontend. The backend is implemented using Flask and is responsible for data processing, automated QC, and data management, while the frontend is implemented in React and provides an interactive interface for visual inspection and user interaction. The backend integrates RDKit [10], a widely used open source cheminformatics toolkit, for structure-based preprocessing tasks such as parsing SMILES strings, calculating exact masses, and generating molecular formulas. Another used library, OpenMS [11] is a robust open source framework for MS data analysis, and the pyOpenMS bindings expose these algorithms within Python. pyOpenMS naturally supports the standard mzML format, aligning with open data standards that are crucial for reproducibility and long-term data access. The framework is built around the principle of combining the computational robustness of Python with the flexibility of a modern web interface, ensuring that both expert users and students can efficiently interact with complex HRMS datasets. Collectively, these features allow pyMSscreen to bridge the gap between restrictive commercial solutions and other open source packages, offering a versatile and user-friendly environment for QC in HRMS. Selected case studies show representative functionality where pyMSscreen could be used to complement current workflows.

## Implementation

pyMSscreen combines pre-screening and automated QC within a single, modern framework with Python Flask as the backbone, offering access to a variety of scientific and cheminformatics libraries like pyOpenMS [12] for data processing and RDKit for structural handling [10]. The frontend is built with React and integrates Plotly.js [13] offering a responsive interface for real-time interaction with results and intuitive data visualisation. Compared to its predecessor Shinyscreen [8], this new framework is designed to improve computational efficiency and analytical flexibility, while maintaining ease of use. Its modular architecture ensures compatibility across operating systems and computational infrastructures, making it broadly applicable to target, suspect screening and NTS workflows. These are the main modes of HRMS data interpretation, each requiring distinct levels of prior mass or structural information and QC. To support NTS workflows, pyMSscreen operates on feature lists of masses generated during prior feature extraction and peak-picking workflows using external software, where the detected features remain unknown. pyMSscreen was designed to support all three modes through a unified QC approach, enabling consistent evaluation of MS^1^ intensity, retention time (RT) alignment of MS^2^ scans, and noise detection, irrespective of the starting information.

**Fig. 1:**
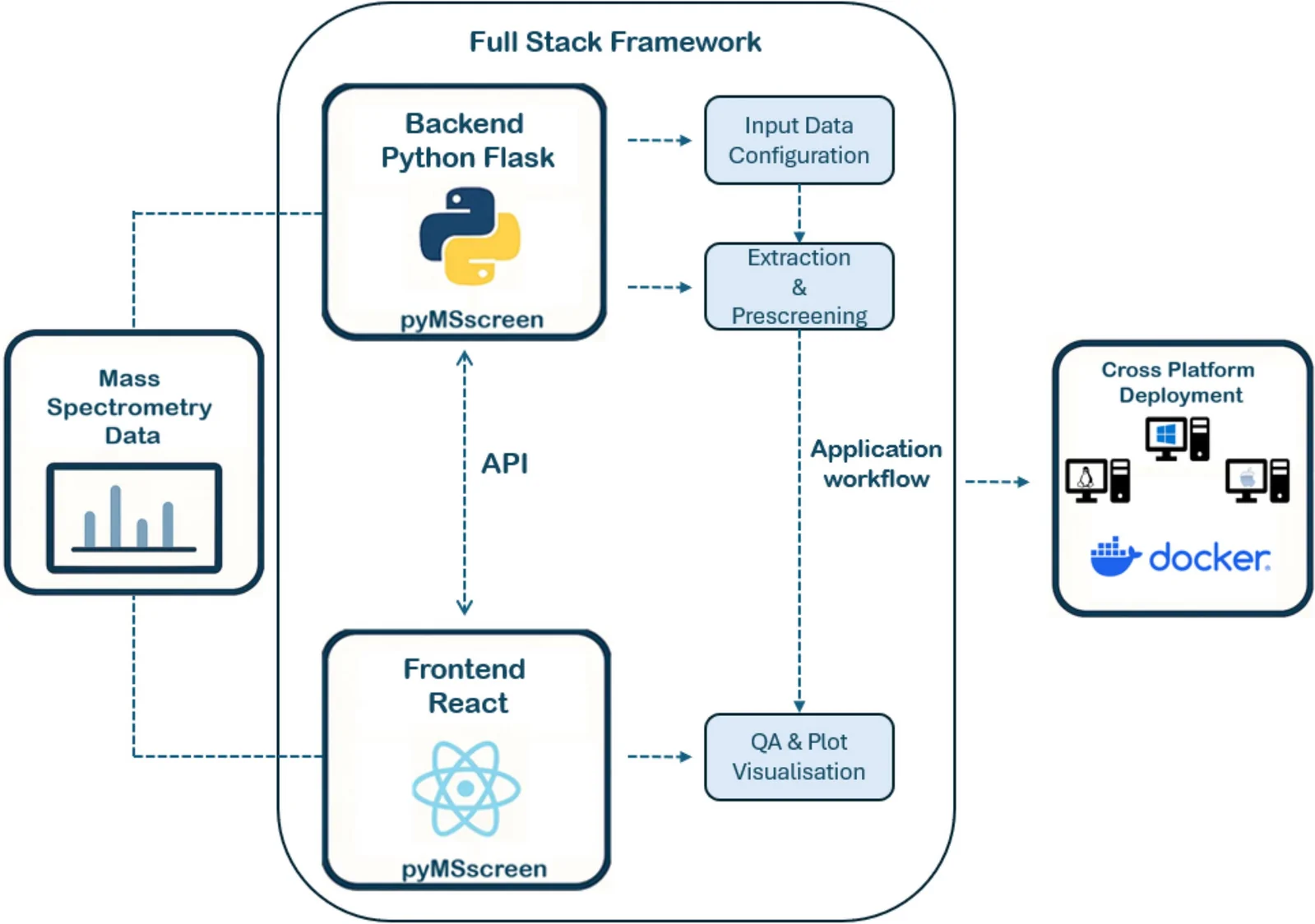
pyMSscreen—full stack framework and application workflow

Figure 1 illustrates the operational workflow of the full-stack framework. Experimental data in mzML format is ingested by the backend, where the Flask application orchestrates input configuration and initiates signal extraction and prescreening using pyOpenMS and RDKit. The processed results are exposed through a RESTful application programming interface (API) and retrieved by the React frontend, which manages quality assessment and renders chromatographic and spectral visualisations with Plotly.js [13]. The complete environment is distributed within Docker containers, ensuring reproducible execution and consistent deployment across heterogeneous computing platforms.

### Software architecture

#### Backend layer

The server side is built with Flask, a lightweight Python web framework for rapid development of RESTful APIs. Flask’s minimalist “microframework” design provides essential functions (routing and HTTP handling) without excessive overhead, making it easy to integrate scientific libraries. All core functionality is exposed through well-defined REST API endpoints, keeping the backend stateless and client-agnostic. Structure-based preprocessing is performed using RDKit [10], including parsing of SMILES strings, calculation of exact masses, and generation of molecular formulas The resulting values are then combined to construct a comprehensive compound list, which guides subsequent spectral extraction. Configuration files (inputs and settings) are serialized in human-readable YAML and CSV formats to ensure reproducibility and interoperability with other tools.

For HRMS data processing, the backend leverages pyOpenMS (the Python bindings for the OpenMS C +  + library) to parse mzML files and extract spectral information. Thus, the backend’s MS data processing pipeline combines OpenMS’s powerful algorithms with the approachability of Python.

#### Frontend layer

The frontend is implemented in React, a JavaScript library for building interactive user interfaces. React enables a dynamic single page application that responds immediately to user input without requiring full page reloads. The interface is organised into modular components aligned with the application workflow. Core modules include data setup for uploading mzML files and compound lists, extraction and prescreening configuration for adjusting processing parameters, and a visualisation dashboard for plot visualization and manual revision of QC results. This modular structure promotes code reuse and extensibility, allowing common elements such as file upload widgets or compound tables to be deployed across multiple views, while additional functionality can be introduced without modifying existing modules.

React’s declarative paradigm ensures that the interface remains tightly coupled to the analysis state. For example, when extraction is completed, the change in state automatically triggers the rendering of plots and summary tables. State management is achieved through the Context API, which maintains shared information such as working directories, uploaded files, and extracted results. This approach provides consistency across workflow stages while preserving modular independence, allowing users to move seamlessly between tasks with their inputs and results retained throughout the session.

Visualisation of HRMS data is a central feature of pyMSscreen, implemented with Plotly.js [13] through its React integration. Plotly was chosen for its ability to generate interactive, publication-quality plots within the browser. Within the Visualisation Dashboard, users can examine EICs and MS^2^ spectra for each compound in an interactive environment. These plots allow detailed inspection, such as zooming into chromatographic peaks to assess their shape or overlaying multiple EIC traces to compare e.g., data from reference standard mixtures with sample measurements. The peaks are aligned using Plotly’s subplot functionality and each EIC is displayed together with its corresponding MS^2^ spectrum in a stacked view, with synchronised axes to facilitate RT comparison. This is achieved by preparing the plot data in the browser after retrieval from the Flask API and rendering combined layouts through the Plotly interface. Interactivity is further supported by features such as double-clicking legend entries to isolate specific traces and mark the associated peaks. Users can also filter and group plots according to metadata such as sample tag or adduct, which enables focused evaluation of subsets within larger datasets.

A design choice was made to handle configuration (run-specific parameters) and state using simple files in standard formats. A JSON file (state.json) maintains the list of input files and compounds in a given session, allowing the stateless server to reconstruct analysis context on each request. The rationale is that configuration in YAML (for extraction settings like mass tolerances, intensity thresholds, retention time windows, etc.) makes the analysis setup transparent. The users or reviewers can open the extract_config.yaml file and immediately see the exact parameters used for peak picking and QC. This YAML config is written by the backend whenever the user updates settings via the UI (through a /save_extract_config endpoint) and is read back on each analysis run to ensure reproducibility of results. Using file-based JSON/YAML, pyMSscreen remains lightweight and deployable. All state files reside in a single folder that can be zipped for archival or debugging. This approach also aligns with best practices in maintaining human-readable files, allowing the users to directly inspect the input parameters.

### Axios and REST API calls

Communication between the frontend and backend occurs exclusively through HTTP requests. The backend exposes a RESTful API with endpoints corresponding to each key operation in the workflow, and the React frontend interacts with these exclusively through HTTP requests. Axios, a JavaScript client for asynchronous communication, is used to transmit JSON payloads, manage responses, and handle errors. Its compatibility with React’s asynchronous programming model enables concise implementation, and the calls are encapsulated within a dedicated utility module (api.js) to preserve clarity and maintainability of the interface code. The use of Axios across all communication ensures consistency and facilitates global configuration of network parameters. The stateless design of the REST API, in which each request contains all necessary contextual information such as working directory and chemical identifiers, enhances scalability and reliability. Independent requests can be processed in parallel or distributed across computational resources without the need for shared state.

### Deployment

The entire pyMSscreen framework is packaged as a Docker container, which encapsulates all dependencies and environment settings to ensure reproducible installation across platforms. This containerization simplifies deployment on local machines, institutional servers, or cloud infrastructures, and supports both single-user and multi-user setups with minimal configuration. By adopting a full-stack architecture, pyMSscreen overcomes the scalability and extensibility limitations inherent to R/Shiny applications, offering a sustainable solution for both high-throughput workflows and exploratory research.

### Application workflow

**Fig. 2:**
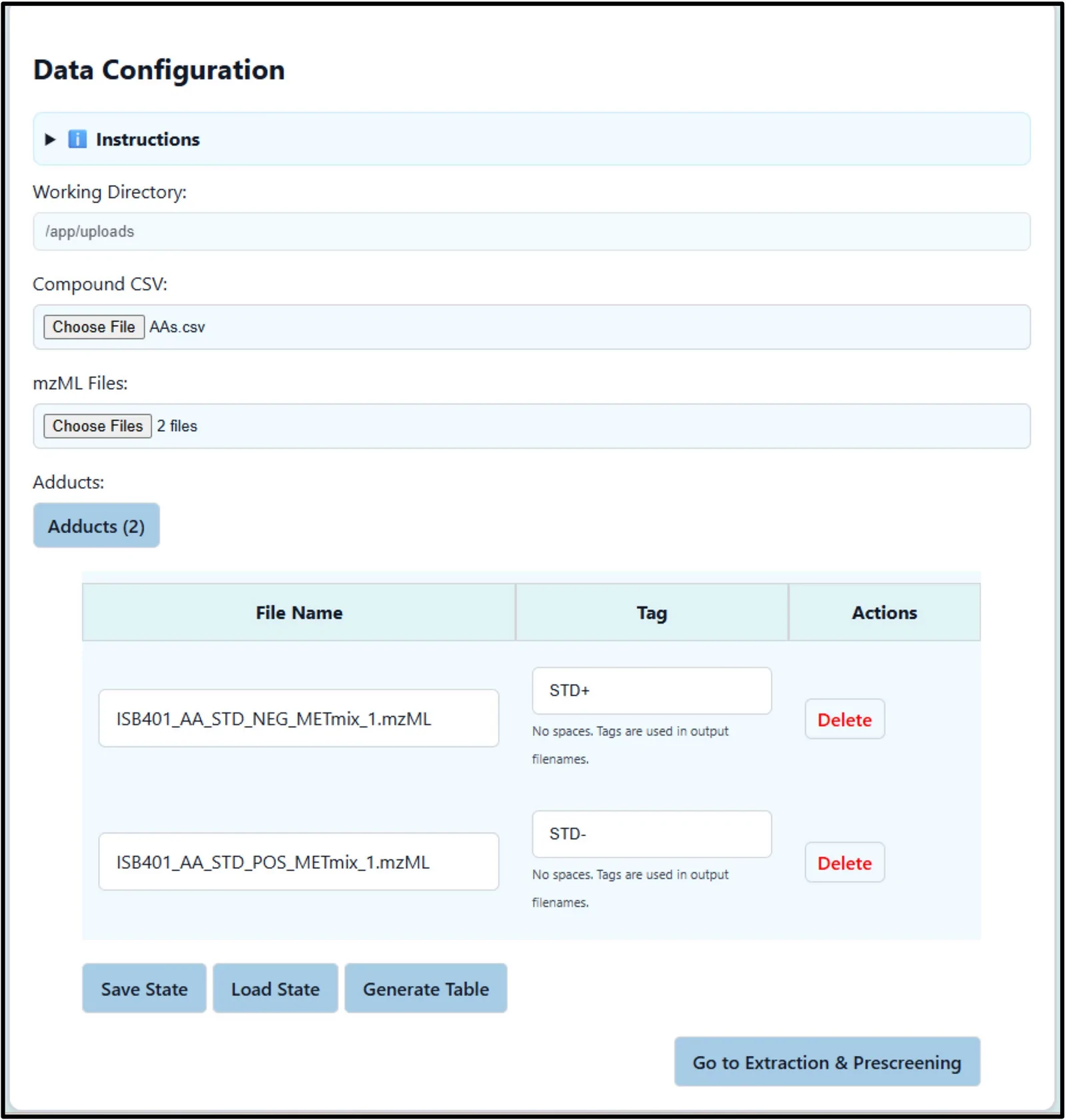
pyMSscreen—user input configuration

The workflow begins with the specification of input data (Fig. 2). The configuration page accepts two resources: (i) a compound list in CSV format and (ii) mass spectrometry data files in mzML format. The compound list can be provided either as a set of precursor *m/z* or neutral exact mass values for NTS (obtained in advance from peak picking output generated by other software e.g., patRoon output) or annotated compound entries including chemical identifiers and SMILES strings for target or suspect screening workflows. An example compound list with supported input fields is provided as a reference input file, with corresponding mzML files on Zenodo. The MS data need to be annotated with sample tags and adducts via the interface. File paths are stored in a state.json file, which defines the working context for all subsequent operations. By default, all inputs and generated outputs are stored in the working directory `/app/uploads`, which is used internally by the application. In the Docker based deployment, this directory is mapped to a user defined location on the host system through a volume mount configured via environment variables specified in the.env file prior to building and starting the docker container. This configuration ensures persistent storage of uploaded data and generated results outside the container across restarts and updates. Further details are explained in the README.

The backend validates SMILES strings (where present in the compound list) and uses RDKit to calculate exact masses and molecular formulas. Adducts are selected from a drop-down menu in the application, and the corresponding mass-to-charge ratios are calculated for each mzML file for the target and suspect screening workflows. The results are compiled into a comprehensive table (comprehensive_table.csv) that links structural information with analytical metadata and provides the basis for spectral extraction.

**Fig. 3:**
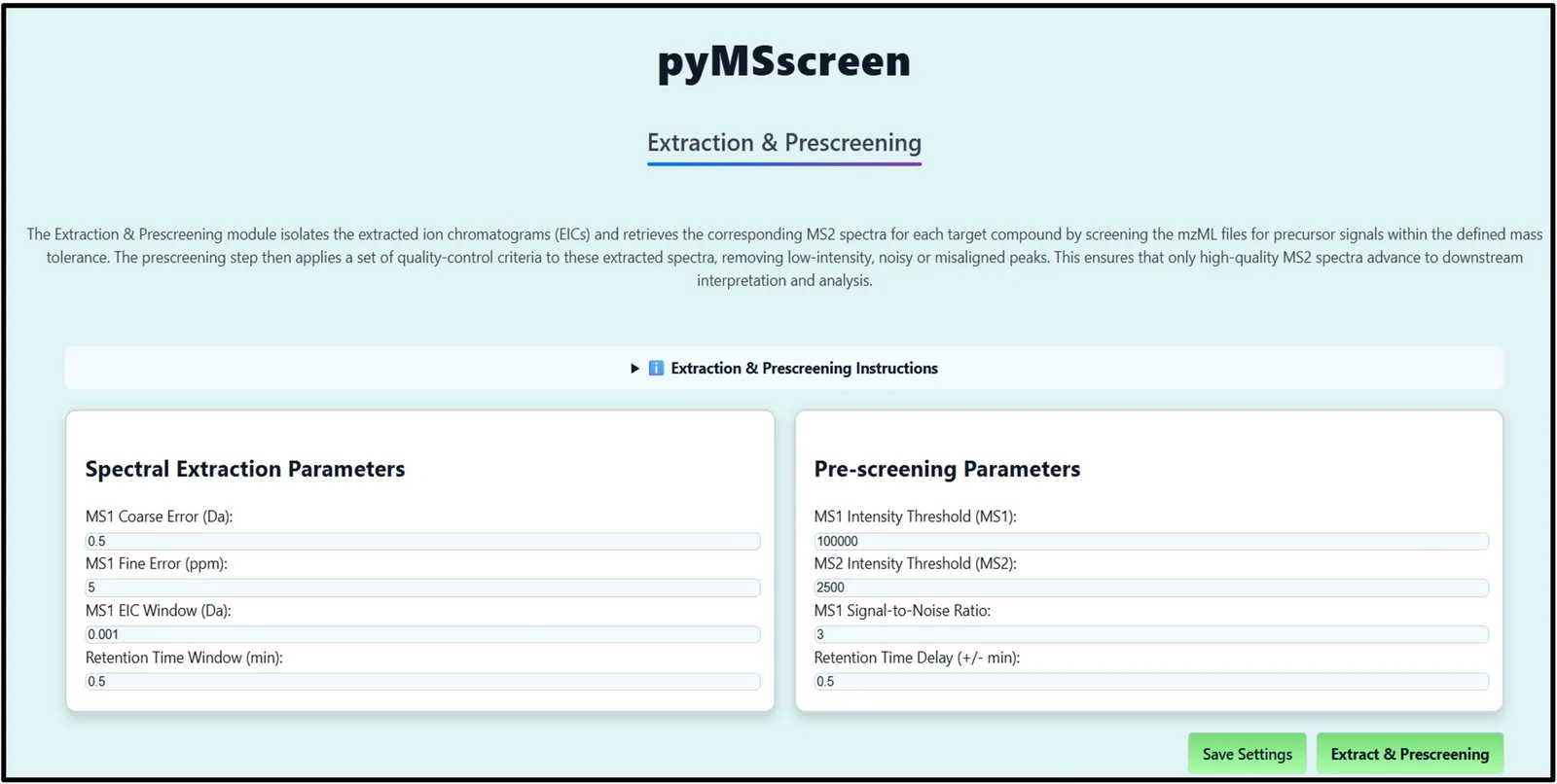
Extraction and prescreening parameters

Spectral extraction is carried out with the pyOpenMS library. Each mzML file is loaded into an MSExperiment object, which is separated into MS^1^ and MS^2^ scans. For every mass, EICs are constructed by summing intensities within the specified mass tolerance across MS^1^ scans, while MS^2^ spectra are simultaneously screened for precursor ions within a defined tolerance (Fig. 3). For spectra that match, fragment lists, total intensities, and retention times are recorded. The resulting spectral data are exported as CSV files for downstream inspection. For visualization and peak prioritization, EICs are evaluated using a local maximum based peak detection approach with a 5-point moving average window, a minimum peak prominence of 5% of the maximum EIC intensity, a minimum chromatographic separation of 0.15 min, and a minimum peak width of 3 consecutive data points. The peak selection criteria help reduce noise and avoid closely overlapping or low intensity signals, while retaining the most representative chromatographic features within the EIC. In cases where multiple chromatographic signals are present within the same EIC, the most intense signal is prioritized for automated QC evaluation, while the top three most intense chromatographic peaks are retained and visualized to support manual inspection of chromatographic complexity and potential co-elution effects.

The extracted data are then evaluated by a prescreening module using parameters defined in YAML configuration file. By default, spectral extraction is performed using a precursor mass tolerance of 5 ppm, whereas the prescreening parameters are set to a minimum MS^1^ intensity threshold of 10^5^, a maximum retention time deviation of 0.5 min between corresponding MS1 and MS2 signals, and a signal-to-noise ratio threshold of 3. These parameters can be adjusted by the user depending on the instrument configuration and dataset characteristics (Fig. 3). MS2 spectra are associated with the closest chromatographic peak apex within the specified retention time tolerance window. If no MS2 signal is detected within the defined retention time tolerance, the feature is retained in the summary table but flagged accordingly during QC evaluation. Each criterion produces a binary flag, and these flags collectively determine the overall classification. The results are consolidated into a summary table (summary_table.csv), which can be reviewed and, when necessary, manually adjusted by the user.

**Fig. 4:**
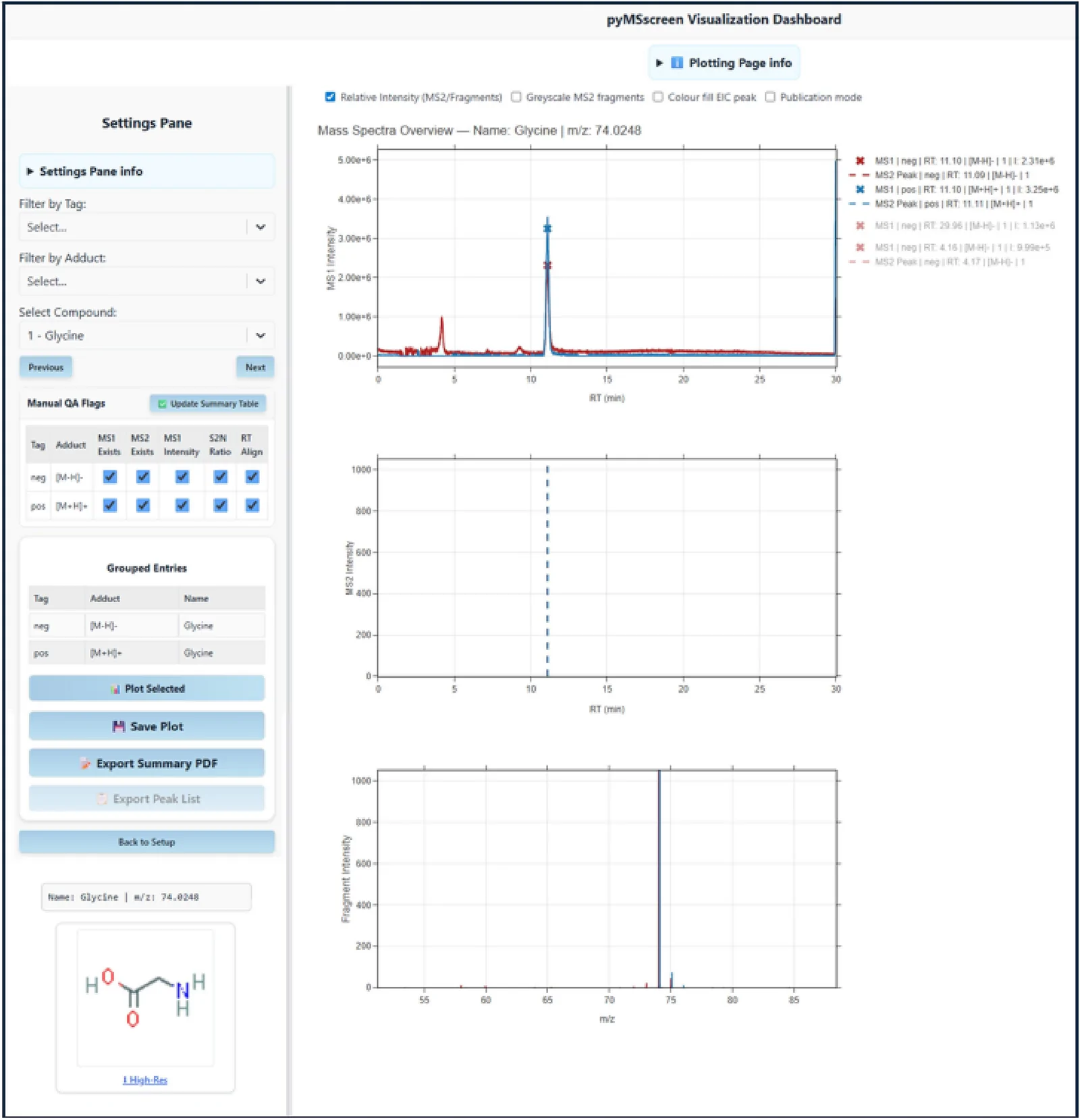
Overview of visualisation dashboard

The visualisation dashboard (Fig. 4) serves the user with the comprehensive compound list and QC summary for client side rendering and streams per compound EIC/MS^2^ CSVs for on demand plotting. The frontend, implemented in React with Plotly.js [13], consumes these endpoints to produce interactive chromatograms, MS^2^ spectra, and fragment distributions in the browser, enabling rapid review without platform specific desktop software. The settings pane includes the option for the user to visualise the Plots filtered per tag.

The summary table gives an overview of different QC flags that the user can check/uncheck after visual inspection of the chromatogram. When a single tag is filtered by using sample and compound tags, the Export Peak List button is enabled for the users to copy the MS^2^ peak list to be copied to the clipboard which can further be used for subsequent workflows, for e.g., in silico spectral matching using MetFrag [14]. The Plotting pane represents the EIC, MS^2^ scans and MS^2^ fragments as subplots. The legend also marks the trace which can be selected by double clicking. This allows the user to manually do QC after inspecting the plots and the RT information. The interactive plots can be saved as individual plots based on the current selection or a summary of plots can be downloaded by clicking the Export Summary PDF button.

## Results and discussion

The performance of pyMSscreen is demonstrated using two representative use cases. The first use case covers a workflow applied during teaching activities, where expected amino acids can be identified in reference standards (to determine experimental RTs) then screened in yeast samples to assess changes in intensity in different scenarios. pyMSscreen was used to perform automated QC of the corresponding chromatographic peaks, including assessment of peak shape, signal intensity, RT consistency, and background noise and compared with MZmine 4.8.0 [5]. This use case was designed to assess the suitability of pyMSscreen for systematic QC and provides the basis for subsequent evaluation of processing speed relative to vendor-based software, Xcalibur [3]. The second use case demonstrates the application of pyMSscreen within a suspect screening workflow using the surface water dataset from Aurich et al. [15], where candidates were annotated using patRoon (version 2.3) and subsequently evaluated with pyMSscreen for chromatographic quality and signal reliability. This example illustrates how chromatographic QC can be integrated downstream of suspect annotation to support the interpretation of suspect screening results and identify potential peak-picking artefacts.

### Use case 1: Target and non-target screening of amino acids in yeast metabolites

Two batches of the yeast strain FY4 wild-type (WT) and the DLD3 knockout (KO) mutant, a model for D-2-hydroxyglutaric aciduria, were analyzed. This mutation alters levels of α-ketoglutarate, D-2-hydroxyglutarate, and several other cofactors. The analysis of amino acids in these strains provides a critical snapshot of the downstream metabolic shifts caused by this mutation. Both strains were cultured under identical conditions and normalized to an optical density of 2 prior to extraction. Data were acquired using standard LC Glasgow method [16] and a Q Exactive^™^ HF mass spectrometer (Thermo Scientific) operated in both positive ( +) and negative ( −) electrospray ionization modes. Raw files were converted to mzML format for subsequent analysis using Proteowizard (version 3.0.21075) [17].

**Fig. 5:**
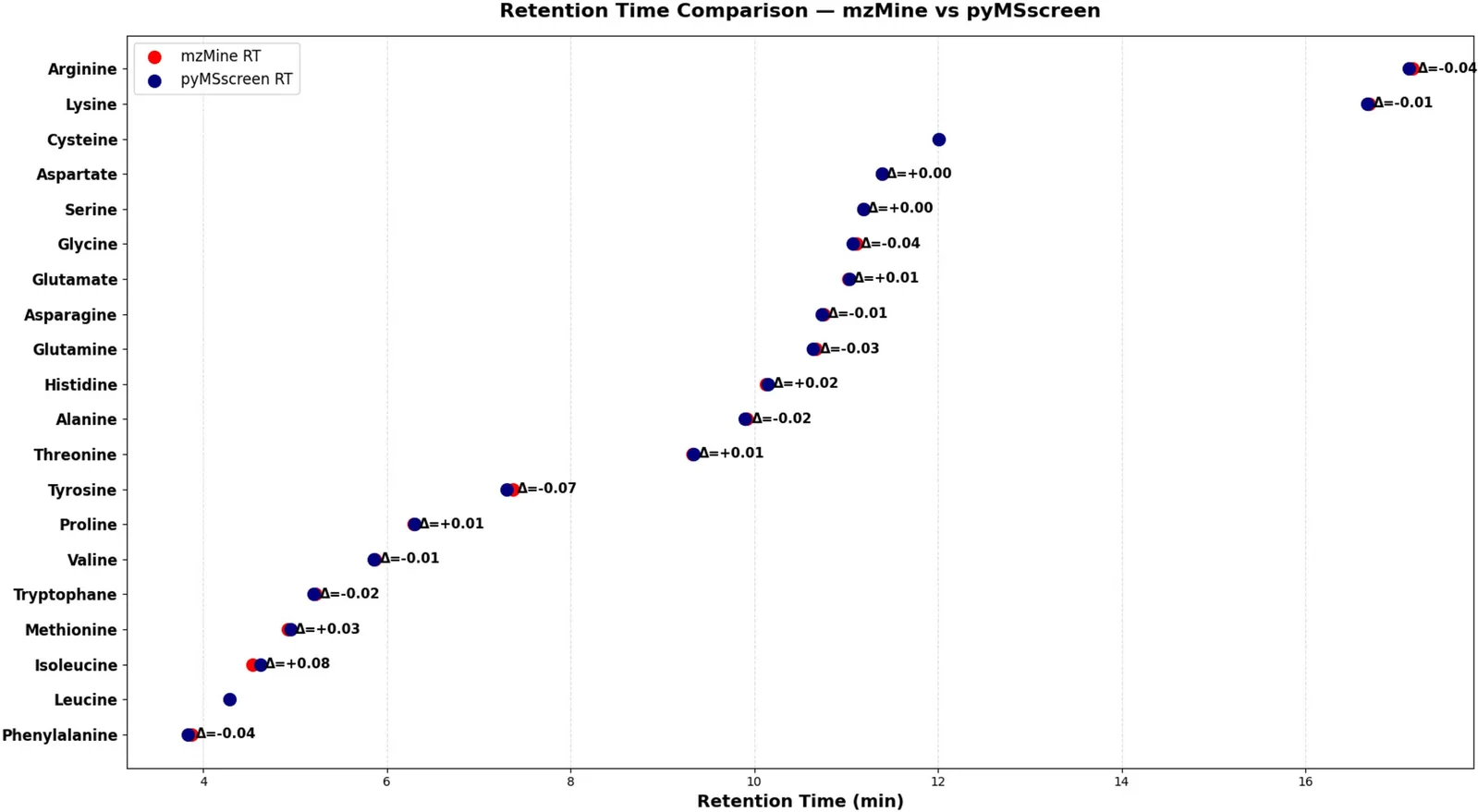
Retention time comparison between pyMSscreen and MZmine

Twenty proteinogenic amino acids were evaluated in both positive and negative ionization modes using a target input list containing SMILES and Name entries for each compound. RT and intensity obtained with pyMSscreen were compared with those from the non-target workflow in MZmine 4.8.0. as shown in Fig. 5.

pyMSscreen detected all 20 amino acids across the dataset while MZmine detected 18 of the 20 amino acids. For compounds detected by both tools, RTs were highly consistent, indicating comparable chromatographic alignment (Fig. 5). The two amino acids not reported by MZmine were observable in the EIC generated by pyMSscreen, highlighting the benefit of interactive visual inspection of the chromatographic peaks.

**Fig. 6:**
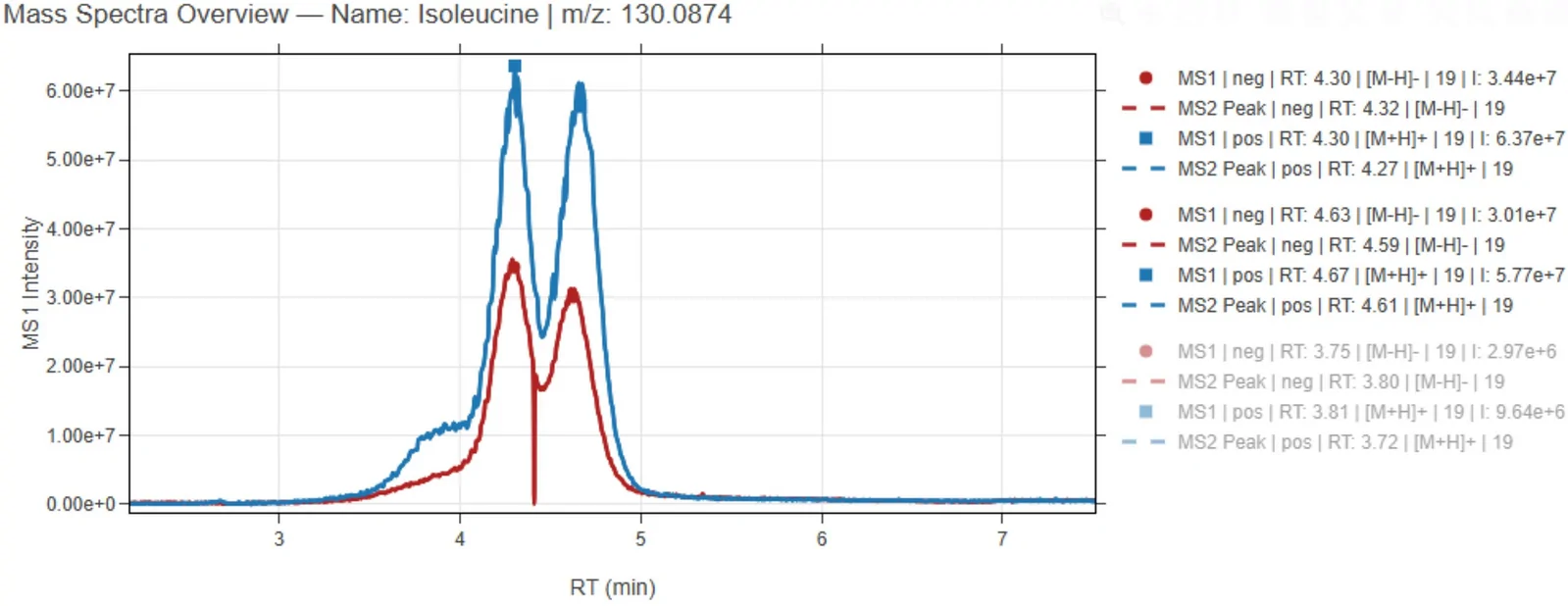
EIC for m/z 132.1019 showing two peaks at approximately 4.3 and 4.6 min, consistent with the presence of the isomeric amino acids leucine and isoleucine

An illustrative example is provided by the isomeric amino acids leucine and isoleucine, which share the same exact mass and therefore produce identical precursor *m/z* values. Isoleucine was not detected by MZmine due to its close elution with leucine. In the EIC for *m/z* 132.1019 generated by pyMSscreen, two closely eluting peaks are clearly visible at approximately 4.3 and 4.6 min (Fig. 6). Comparison with individually analyzed standards enabled unambiguous RT based assignment of the earlier peak (4.3 min) to leucine and the later peak (4.6 min) to isoleucine. The visualization dashboard further enables direct inspection of the associated MS^1^ and MS^2^ acquisitions for each peak. During automated processing, the algorithm prioritizes the most intense chromatographic signal for downstream evaluation while retaining the top three most intense peaks within the chromatogram. This approach transparently displays the underlying chromatographic complexity and supports QC by enabling inspection of co elution effects, while still preserving access to lower intensity but analytically relevant features.

Beyond improvements in feature interpretability, pyMSscreen minimizes manual processing associated with vendor-based workflows by integrating chromatographic inspection, MS^1^/MS^2^ evaluation, and QC within a single web interface. pyMSscreen was compared against the standard vendor-based workflow for this dataset (Thermo Scientific^™^ Xcalibur^™^). In Thermo Xcalibur, chromatographic data extraction can be performed using Quan Browser by manually defining target *m/z* values and retention time windows in the processing setup, after which multiple files can be processed together. Once a processing method is established, the chromatographic peaks are integrated automatically and can be manually inspected for each file and compound. Qual Browser additionally allows inspection of chromatographic traces and MS^1^ and MS^2^ spectra across multiple files by creating templates. However, the setup and management of processing methods and templates can be time consuming, particularly when working with large suspect lists or when iterative updates are required. In addition, systematic inspection of MS^2^ spectral quality and its alignment is not fully integrated within a single workflow.

In contrast, pyMSscreen automates chromatographic extraction across all files and ionization modes using a compound file, thereby removing the need for manual configuration of processing methods or templates. All data files are processed simultaneously in a single run, reducing user input and enabling rapid analysis of complete datasets. The pyMSscreen visualisation dashboard integrates chromatographic and spectral review within a browser based environment, allowing users to filter samples by metadata, isolate individual chromatographic traces, and inspect MS^1^ and MS^2^ spectra within a unified interface. This allows focused inspection of individual samples within large datasets, making the review process considerably faster and more consistent.

Overall, this use case demonstrates that pyMSscreen serves as an effective visualization tool that enables rapid determination of accurate retention times for target compounds and facilitates efficient QC. For the dataset used in this study (6 mzML files and 20 amino acids), automated QC in pyMSscreen was completed in less than 2 min. Based on iterative evaluation across different user groups, pyMSscreen complements comprehensive workflows such as MZmine by enabling plot generation and supporting QC through an interactive web-based interface. By reducing the need for manual inspection across multiple data files, the use of pyMSscreen improves the efficiency of data processing in HRMS workflows.

### Use case 2: NTS of surface water

To further demonstrate the performance of pyMSscreen in NTS, the QC evaluation was applied to the June 2020 surface water dataset from Aurich et al. [15]. In the original study, patRoon was used to annotate features using MS^1^ detection, spectral libraries, and diagnostic fragments. Candidate compound structures were ranked by MS^2^ spectral similarity scores and assigned identification confidence to Levels 2, 3a, and 3b following an adapted scheme by Talavera Andújar et al. [18], which was based on confidence levels defined in 2014 [19]. Specifically, Level 2 corresponds to a good MS^2^ library match with a MassBank of North America (MoNA) similarity score of atleast 0.9, Level 3a to a fair match with MoNA scores between 0.7 and 0.9, and Level 3b defined with MonA scores between 0.4 and 0.7 (described further in [15]). These identification levels were retained unchanged in the present study to ensure consistency with the original dataset.

**Fig. 7:**
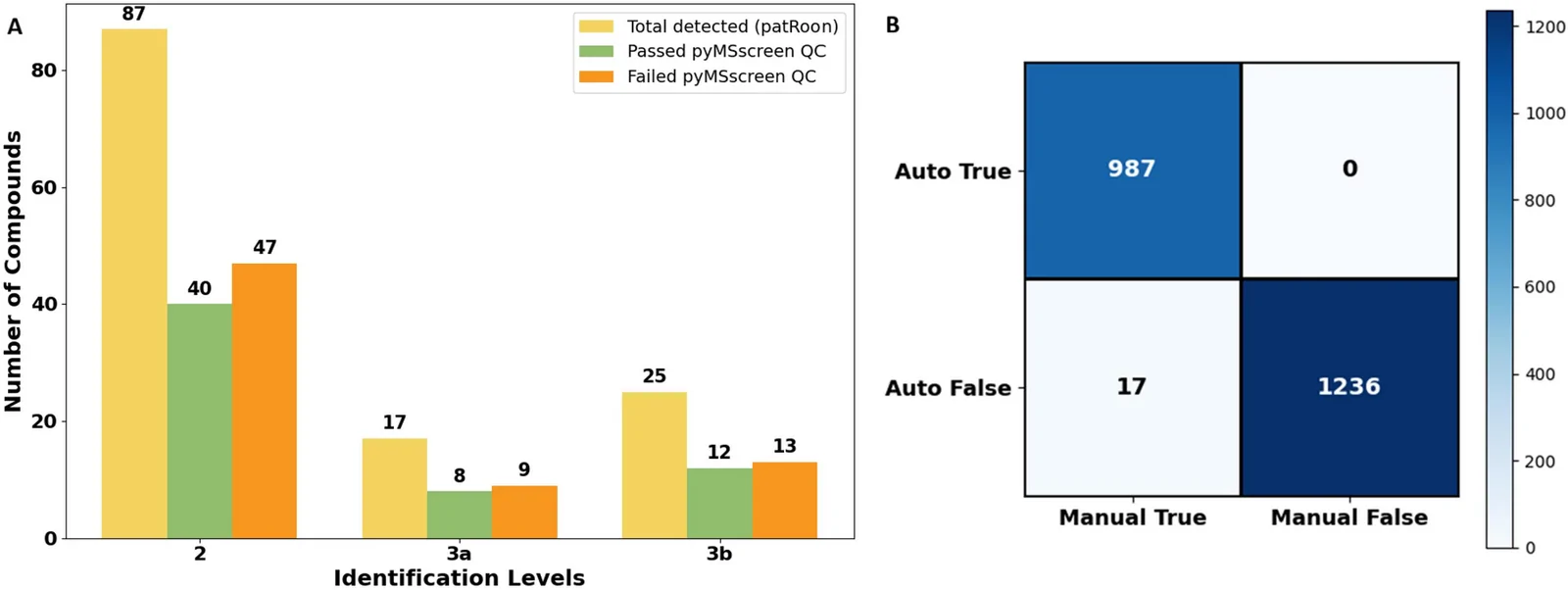
**a** pyMSscreen QC outcome on patRoon annotated compounds. **b** pyMSscreen QC consistency with user-validated peak quality assessment

While this approach is powerful for assigning plausible structures, patRoon does not directly assess chromatographic signal quality, which is critical for confirming whether annotated features represent robust detections. To address this, all features annotated as Level 2, 3a and 3b were reevaluated using pyMSscreen’s built-in QC module. pyMSscreen applies standardized rules for QC using instrument-specific thresholds, in this case MS^1^ intensity > 10^5^, RT window between MS^1^ and MS^2^ should be within 0.5 min, and the signal to noise ratio is set to be 3. By evaluating features without prior blank subtraction, the pyMSscreen workflow provides a transparent assessment of chromatographic quality, ensuring that QC metrics reflect the actual spectral data rather than processing artifacts. Figure 7a illustrates significant variability in chromatographic quality across all identification levels. While patRoon provides plausible structural assignments, the subsequent QC analysis showed that only circa 50% of these features passed the spectral and intensity thresholds. This discrepancy demonstrates that automated workflows alone are insufficient for robust feature validation, as it may include signals that lack the chromatographic integrity required for reliable quantification.

pyMSscreen also supports a semi-automated workflow in which users can visually inspect chromatographic peaks and manually override QC flags where justified. This provides a practical balance between automation and expert oversight, particularly valuable for ambiguous or borderline chromatographic features. To assess the reliability and flexibility of pyMSscreen’s QC framework, the fully automated QC output was compared with the manual review of QC flags directly within the application. The default automated QC flags (“auto QC”) were compared with the user-reviewed QC annotations (“semi-automated QC”), which represent the final curated decisions made after visual inspection within pyMSscreen itself. This comparison does not constitute external manual validation, but rather evaluates how well the automated rules agree with expert decisions when both are embedded in the same workflow environment.

The resulting confusion matrix (Fig. 7b) demonstrates a high level of concordance between automated and user-validated QC outcomes. pyMSscreen’s automated QC correctly matched the user’s final decision for 2223 out of 2240 features. Specifically, the automated QC accepted 987 peaks that were also confirmed by user inspection (true positives) and rejected 1236 features that were also rejected after manual review (true negatives). Notably, the automated algorithm produced no false positives (FP = 0), meaning it never approved a peak that the user subsequently deemed unacceptable. Only 17 features initially rejected by the automated QC were later accepted upon user review (false negatives); these typically displayed borderline peak morphology judged acceptable after visual inspection.

The automated QC achieved perfect precision (1.00) and specificity (1.00), indicating that the rule-based system does not falsely classify poor-quality features as acceptable. Accuracy was 0.992, reflecting strong overall agreement between automated and user-validated QC decisions. The recall of 0.983 reflects pyMSscreen’s intentionally conservative QC behavior: while it occasionally flags borderline peaks that users later deem acceptable, it never incorrectly passes poor-quality signals, based on the default settings used here, which are selected based on instrument knowledge.

This evaluation highlights the complementary strengths of fully automated and semi-automated modes in pyMSscreen. The automated QC provides a rigorous, reproducible baseline that eliminates false positives and enforces strict chromatographic standards. At the same time, the user-adjustable interface allows domain experts to override automated decisions when appropriate, supporting a flexible, semi-automated workflow. Together, these features make pyMSscreen a robust and transparent QC framework that enhances confidence in suspect and non-target screening results.

This use case illustrates that pyMSscreen provides an essential QC complement to patRoon. While patRoon efficiently generates structurally plausible annotations, pyMSscreen helps identify which of these annotations correspond to true, high-quality chromatographic features. When combined, the two tools form a more reliable and reproducible workflow for NTS studies.

## Conclusion

pyMSscreen establishes a unified and transparent framework for automated QC and visualization of HRMS data. By combining the computational strength of Python with a flexible React-based frontend, the application delivers an accessible yet powerful environment for both target, suspect and non-target screening applications. Its modular architecture and containerized deployment make it reproducible, scalable, and easily adaptable across research environments. Beyond usability, pyMSscreen introduces a comprehensive QC framework that evaluates MS^1^ peak characteristics, MS^2^ peak alignment, RT consistency, and noise behavior in a transparent and reproducible manner. The development of pyMSscreen was informed by the pros and cons of its predecessor, Shinyscreen, which demonstrated the value of an intuitive graphical interface for HRMS data review. While Shinyscreen substantially improved accessibility, its reliance on the R/Shiny ecosystem imposed important limitations in scalability, multi-user performance, and extensibility. pyMSscreen addresses these issues through a full-stack implementation that decouples data processing from visualization, thus enabling more efficient computation, interactive browser-based review, and seamless integration into diverse research environments.

Future refinement of pyMSscreen will focus on the inclusion of blank evaluation to improve background discrimination and the robustness of QC metrics, alongside the potential addition of automated tag detection and peak quality scoring to support feature prioritization. In addition, planned extensions include an identification module with a focus on evaluation of QC metrics, supporting more informed feature prioritization in large datasets. The React frontend is well suited for embedding interactive predictive components, enabling dynamic feedback between computational inference and expert validation. Such developments will allow pyMSscreen to evolve from a rule-based QC framework into a learning system capable of supporting semi-automated annotation, accelerating data-driven discovery in metabolomics, environmental analysis, and cheminformatics. It bridges the gap between restrictive commercial tools and complex open source pipelines, offering a future-ready platform for robust and efficient HRMS screening.

## Data Availability

The code for pyMSscreen can be found at https://gitlab.com/uniluxembourg/lcsb/eci/pymsscreen under the Artistic License 2.0. All resources and documentation are provided under open licenses, ensuring accessibility and reproducibility. The datasets analysed in the Use Case 1 are publicly available in mzML format via Zenodo at https://zenodo.org/records/14950291. The datasets analysed in the Use Case 2 are publically available as dataset MSV000092221 from the GNPS MassIVE repository, accessible via ftp://massive.ucsd.edu/MSV000092221/ [15]. The corresponding compound input lists for both the Use Cases can be found at https://gitlab.com/uniluxembourg/lcsb/eci/pymsscreen/-/blob/main/example_inputs.

## Declarations

### Availability and requirements

• Project name: pyMSscreen

• Project home page: https://gitlab.com/uniluxembourg/lcsb/eci/pymsscreen

• Operating system(s): Platform independent

• Programming language: JavaScript (63%), Python (33 %), other (3 %)

• Other requirements: pyOpenMS

• License: Artistic 2.0

• Any restrictions to use by non-academics: None
